# Classification of G-protein coupled receptors based on support vector machine with maximum relevance minimum redundancy and genetic algorithm

**DOI:** 10.1186/1471-2105-11-325

**Published:** 2010-06-16

**Authors:** Zhanchao Li, Xuan Zhou, Zong Dai, Xiaoyong Zou

**Affiliations:** 1 School of Chemistry and Chemical Engineering, Sun Yat-Sen University, Guangzhou 510275, PR China

## Abstract

**Background:**

Because a priori knowledge about function of G protein-coupled receptors (GPCRs) can provide useful information to pharmaceutical research, the determination of their function is a quite meaningful topic in protein science. However, with the rapid increase of GPCRs sequences entering into databanks, the gap between the number of known sequence and the number of known function is widening rapidly, and it is both time-consuming and expensive to determine their function based only on experimental techniques. Therefore, it is vitally significant to develop a computational method for quick and accurate classification of GPCRs.

**Results:**

In this study, a novel three-layer predictor based on support vector machine (SVM) and feature selection is developed for predicting and classifying GPCRs directly from amino acid sequence data. The maximum relevance minimum redundancy (mRMR) is applied to pre-evaluate features with discriminative information while genetic algorithm (GA) is utilized to find the optimized feature subsets. SVM is used for the construction of classification models. The overall accuracy with three-layer predictor at levels of superfamily, family and subfamily are obtained by cross-validation test on two non-redundant dataset. The results are about 0.5% to 16% higher than those of GPCR-CA and GPCRPred.

**Conclusion:**

The results with high success rates indicate that the proposed predictor is a useful automated tool in predicting GPCRs. GPCR-SVMFS, a corresponding executable program for GPCRs prediction and classification, can be acquired freely on request from the authors.

## Background

G protein-coupled receptors (GPCRs), also known as 7 α-helices transmembrane receptors due to their characteristic configuration of an anticlockwise bundle of 7 transmembrane α helices [1], are one of the largest superfamily of membrane proteins and play an extremely important role in transducing extracellular signals across the cell membrane via guanine-binding proteins (G-proteins) with high specificity and sensitivity [2]. GPCRs regulate many basic physicochemical processes contained in a cellular signaling network, such as smell, taste, vision, secretion, neurotransmission, metabolism, cellular differentiation and growth, inflammatory and immune response [3-9]. For these reasons, GPCRs have been the most important and common targets for pharmacological intervention. At present, about 30% of drugs available on the market act through GPCRs. However, detailed information about the structure and function of GPCRs are deficient for structure-based drug design, because the determination of their structure and functional using experimental approach is both time-consuming and expensive.

As membrane proteins, GPCRs are very difficult to crystallize and most of them will not dissolve in normal solvents [10]. Accordingly, the 3 D structure of only squid rhodopsin, β1, β2 adrenergic receptor and the A2A adenosine receptor have been solved to data. In contrast, the amino acid sequences of more than 1000 GPCRs are known with the rapid accumulation of data of new protein sequence produced by the high-throughput sequencing technology. In view of the extremely unbalanced state, it is vitally important to develop a computational method that can fast and accurately predict the structure and function of GPCRs from sequence information.

Actually, many predictive methods have been developed, which in general, can be roughly divided into three categories. The first one is proteochemometric approach developed by Lapinsh [11]. However, the methods need structural information of organic compounds. The second one is based on similarity searches using primary database search tools (e.g. BLAST, FASTA) and such database searches coupled with searches of pattern databases (PRINTS) [12]. However, they do not seem to be sufficiently successful for comprehensive functional identification of GPCRs, since GPCRs make up a highly divergent family, and even when they are grouped according to similarity of function, their sequences share strikingly little homology or similarity to each other [13]. The third one is based on statistical and machine learning method, including support vector machines (SVM) [8,14-17], hidden Markov models (HMMs) [1,3,6,18], covariant discriminant (CD) [7,11,19,20], nearest neighbor (NN) [2,21] and other techniques [13,22-24].

Among them, SVM that is based on statistical learning theory has been extensively used to solve various biological problems, such as protein secondary structure [25,26], subcellular localization [27,28], membrane protein types [29], due to its attractive features including scalability, absence of local minima and ability to condense information contained in the training set. In SVM, an initial step to transform protein sequence into a fixed length feature vector is essential because SVM can not be directly applied to amino acid sequences with different length. Two commonly used feature vectors to predict GPCRs functional classes are amino acid composition (AAC) and dipeptide composition (DipC) [2,7,10,16,19,20,22], where every protein is represented by 20 or 400 discrete numbers. Obviously, if one uses AAC or DipC to represent a protein, many important information associated with the sequence order will be lost. To take into account the information, the so-called pseudo amino acid composition (PseAA) was proposed [30] and has been widely used to GPCRs and other attributes of protein studies [10,31-36]. However, the existing methods were established only based on a single feature-set. And, few works tried to research the relationship between features and the functional classes of protein [37-39], or to find the informative features which contribute most to discriminate functional types. Karchin et al [8] also indicated that the performance of SVM could be further improved by using feature vector that posses the most discriminative information. Therefore, feature selection should be used for accurate SVM classification.

Feature selection, also known as variable selection or attribute selection, is the technique commonly used in machine learning and has played an important role in bioinformatics studies. It can be employed along with classifier construction to avoid over-fitting, to generate more reliable classifier and to provide more insight into the underlying causal relationships [40]. The technique has been greatly applied to the field of microarray and mass spectra (MS) analysis [41-50], which has a great challenge for computational techniques due to their high dimensionality. However, there is still few works utilizing feature selection in GPCRs prediction to obtain the most informative features or to improve the prediction accuracy.

So, a new predictor combining feature selection and support vector machine is proposed for the identification and classification of GPCRs at the three levels of superfamily, family and subfamily. In every level, minimum redundancy maximum relevance (mRMR) [51] is utilized to pre-evaluate features with discriminative information. After that, to further improve the prediction accuracy and to obtain the most important features, genetic algorithms (GA) [52] is applied to feature selection. Finally, three models based on SVM are constructed and used to identify whether a query protein is GPCR and which family or subfamily the protein belongs to. The prediction quality evaluated on a non-redundant dataset by the jackknife cross-validation test exhibited significant improvement compared with published results.

## Methods

### Dataset

As is well-known, sequence similarity in dataset has an important effect on the prediction accuracy, i.e. accuracy will be overestimated when using high similarity protein sequence. Thus, in order to disinterestedly test current method and facilitate to compare with other existing approaches, the dataset constructed by Xiao [10] is used as the working dataset. The similarity in the dataset is less than 40%. The dataset contains 730 protein sequences that can be classified into two parts: 365 non-GPCRs and 365 GPCRs. The 365 GPCRs can be divided into 6 families: 232 rhodopsin-like, 44 metabotropic glutamate/pheromone, 39 secretin-like, 23 fungal pheromone, 17 frizzled/smoothened and 10 cAMP receptor. For rhodopsin-like of GPCRs, we further partitioned into 15 subfamilies based on GPCRDB (release 10.0) [53], including 46 amine, 72 peptide, 2 hormone, 17 rhodopsin, 19 olfactory, 7 prostanoid, 13 nucleotide, 2 cannabinoid, 1 platelet activating factor, 2 gonadotropin-releasing hormone, 3 thyrotropin-releasing hormone & secretagogue, 2 melatonin, 9 viral, 4 lysosphingolipid, 2 leukotriene B4 receptor and 31 orphan. Those subfamilies, which the number of proteins is lower than 10, are combined into a class, because they contain too few sequences to have any statistical significance. So, 6 classes (46 amine, 72 peptide, 17 rhodopsin, 19 olfactory, 13 nucleotide and 34 other) are obtained at subfamily level.

### Protein represent

In order to fully characterize protein primary structure, 10 feature vectors are employed to represent the protein sample, including AAC, DipC, normalized Moreau-Broto autocorrelation (NMBAuto), Moran autocorrelation (MAuto), Geary autocorrelation (GAuto), composition (C), transition (T), distribution (D) [54], composition and distribution of hydrophobicity pattern (CHP, DHP). Here 8 and 7 amino acid properties extracted from AAIndex database [55] are selected to compute autocorrelation, C, T and D features, respectively. The properties and definitions of amino acids attributed to each group are shown in Additional file 1 and 2.

According to the theory of Lim [56], 6 kinds of hydrophobicity patterns include: (*i*, *i*+*2*), (*i*, *i*+*2*, *i*+*4*), (*i*, *i*+*3*), (*i*, *i*+*1*, *i*+*4*), (*i*, *i*+*3*, *i*+*4*) and (*i*, *i*+*5*). The patterns (*i*, *i*+*2*) and (*i*, *i*+*2*, *i*+*4*) often appear in the β-sheets while the patterns (*i*, *i*+*3*), (*i*, *i*+*1*, *i*+*4*) and (*i*, *i*+*3*, *i*+*4*) occur more often in α-helices. The pattern (*i*, *i*+*5*) is an extension of the concept of the "helical wheel" or amphipathic α-helix [57]. Seven kinds of amino acids, including Cys (C), Phe (F), Ile (I), Leu (L), Met (M), Val (V) and Trp (W), may occur in the 6 patterns based on the observed of Rose et al [58]. Because transmembrane regions of membrane protein are usually composed of β-sheet and α-helix, CHP and DHP are used to represent protein sequence.

For the pattern (*i*, *i*+*2*), the CHP is computed by Eq. (1):(1)

Where, *N*_(*i,i*+2) _is the number of pattern in position *i *and *i*+*2 *that simultaneously belong to any of 7 kinds of amino acids, and *L *is the sequence length. Other CHP are calculated by using the rule mentioned above. The DHP of pattern (*i*, *i*+*2*), which describes the distribution of the pattern in protein sequence, can be calculated according to Eq. (2):(2)

**Figure 1:**
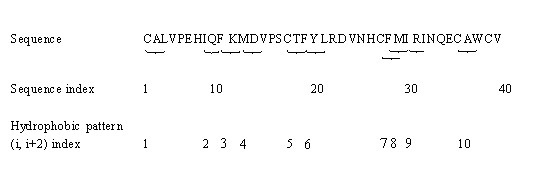
**Random sequence consisting of 40 residues as an example to illustrate derivation of feature vector**.

Where, *S*_(*i,i*+2) _is a feature vector composing of 5 values that are the position in the whole sequence for the first pattern (*i*, *i*+*2*), 25% patterns (*i*, *i*+*2*), 50% patterns (*i*, *i*+*2*), 75% patterns(*i*, *i*+*2*) and 100% patterns (*i*, *i*+*2*), respectively. How to calculate these values is explained below by using a random sequence with 40 amino acids, as shown in Figure 1, which consists of 10 patterns (*i*, *i*+*2*). The 10 patterns (*i*, *i*+*2*) included, CAL (1), IQF (2), FKM (3), MDV (4), CTF (5), FYL (6), CFM (7), FMI (8), IRI (9), CAW (10). The first pattern (*i*, *i*+*2*) is in the pattern position of 1(CAL). The pattern (*i*, *i*+*2*) of (10 × 25% = 2.5≈3) is in the pattern position of 3 (FKM). The pattern (*i*, *i*+*2*) of (10 × 50% = 5) is in the pattern position of 5 (CTF). The pattern (*i*, *i*+*2*) of (10 × 75% = 7.5≈8) is in the pattern position of 8 (FMI). The pattern (*i*, *i*+*2*) of (10 × 100% = 10) is in the pattern position of 10 (CAW). The first letter of the 5 patterns (*i*, *i*+*2*) are C, F, C, F, C, which is corresponding to the residue position of 1, 10, 17, 28, and 36 in the sequence, respectively. Thus, *S*_(*i,i*+2) _= [1 10 17 28 36]. Similarly, the DHP for pattern other than (*i*, *i*+*2*) is also calculated by using the rule.

### The optimized feature subset selection

SVM is one of the most powerful machine learning methods, but it cannot perform automatic feature selection. To overcome this limitation, various feature selection methods were introduced [59,60]. Feature selection methods typically were divided into two categories: filter and wrapper methods. Although filter methods are computationally simple and easily scale to high-dimensional dataset, they ignore the interaction between selected feature and classifier. In contrast, wrapper approaches include the interaction and can also take into account the correlation between features, but they have a higher risk of overfitting than filter techniques and are very computationally intensive, especially if building the classifier has a high computational cost [61]. Considering the characteristics of the two methods, the mRMR belonging to filter methods is used to preselect a feature subset, and then GA belonging to wrapper methods is utilized to obtain the optimized feature subset.

### Minimum redundancy maximum relevance (mRMR)

The mRMR method tries to select a feature subset, each of which has the maximal relevance with target class and the minimal redundancy with other features. The feature subset can be obtained by calculating the mutual information between the features themselves and between the features and the class variables. In the current study, feature is a vector contains 10 type descriptors values of proteins (AAC, DipC, NMBAuto, MAuto, GAuto, C, T, D, CHP and DHP). For binary classification problem, classification variable *l_k _*∈1 or 2. The mutual information *MI(x,y) *of between two features *x *and *y *is computed by Eq.(3):(3)

Where, *p(x_i_,y_j_) *is joint probabilistic density, *p(x_i_) *and *p(y_j_) *is marginal probabilistic density.

Similarly, the mutual information *MI(x,l) *of between classification variable *l *and feature *x *is also calculated by Eq.(4):(4)

The minimum redundancy condition is to minimize the total redundancy of all features selected by Eq.(5):(5)

Where, *S *denoted that the feature subset, and |*S*| is the number of feature in *S*.

The maximum relevance condition is to maximize the total relevance between all features in *S *and classification variable. The condition can be obtained by Eq. (6):(6)

To achieve feature subset, the two conditions should be optimized simultaneously according to Eq. (7):(7)

If continuous features exist in feature set, the feature must be discretized by using "mean ± standard deviation/2" as boundary of the 3 states. The value of feature larger than "mean + standard deviation/2" is transformed to state 1; The value of feature between "mean - standard deviation/2" and "mean + standard deviation/2" is transformed to state 2; The value of feature smaller than "mean - standard deviation/2" is transformed to state 3. In this case, computing mutual information is straightforward, because both joint and marginal probability tables can be estimated by tallying the samples of categorical variables in the data [51]. More explanation about the calculation of probability can be seen from Additional file 3. Detailed depiction of the mRMR method can be found in reference [51], and mRMR program can be obtained from http://penglab.janelia.org/proj/mRMR/index.htm

### Genetic algorithms (GA)

GA can effectively search the interesting space and easily solve complex problems without requiring a prior knowledge about the space and the problem. These advantages of GA make it possible to simultaneously optimize the feature subset and the SVM parameters. The chromosome representations, fitness function, selection, crossover and mutation operator in GA are described in the following sections.

#### Chromosome representation

The chromosome is composed of decimal and binary coding systems, where binary genes are applied to the selection of features and decimal genes are utilized to the optimization of SVM parameters.

#### Fitness function

In this study, two objectives must be simultaneously considered when designing the fitness function. One is to maximize the classification accuracy, and the other is to minimize the number of selected features. The performances of these two objectives can be evaluated by Eq. (8):(8)

Where, *SVM *_ *accuracy *is SVM classification accuracy, *n *is the number of selected features, *N *is the number of overall features.

#### Selection, crossover and mutation operator

Elitist strategy that guarantees chromosome with the highest fitness value is always replicated into the next generation is used to select operation. Once a pair of chromosome is selected for crossover, five random selected positions are assigned to the crossover operator of the binary coding part. The crossover operator was determined according to Eq. (9) and (10) for the decimal coding part, where *p *is the random number of (0, 1).(9)(10)

The method based on chaos [62] is applied to the mutation operator of decimal coding. Mutation to the part of binary coding is the same as traditional GA.

The population size of GA is 30, and the termination condition is that the generation numbers reach 10000. A detailed depiction of the GA can be reference to our previous works [63].

### Model construction and assessment of performance

For the present SVM, the publicly available LIBSVM software [64] is used to construct the classifier with the radial basis function as the kernel. Ten-fold cross-validation test is used to examine a predictor for its effectiveness. In the 10-fold cross-validation, the dataset is divided randomly into 10 equally sized subsets. The training and testing are carried out 10 times, each time using one distinct subset for testing and the remaining 9 subsets for training.

Classifying GPCRs in superfamily level can be formulated as a binary classification problem, namely each protein can be classified as either GPCRs or non-GPCRs. So, the performance of classifier are measured in terms of sensitivity (*Sen*), specificity (*Spe*), accuracy (*Acc*) and Matthew's correlation coefficient (*MCC*) [65], and are given by Eqs. (11)-(14).(11)(12)(13)(14)

Here, *TP*, *TN*, *FP *and *FN *are the numbers of true positives, true negatives, false positives and false negatives, respectively.

The classification of GPCRs into its families and subfamilies is a multi-class classification problem, namely a given protein can be classified into specific family or subfamily. The simple solution is to reduce the multi-classification to a series of binary classifications. We adopted the one-versus-one strategy to transfer it into a series of two-class problems. The overall accuracy (*Q*) and accuracy (*Q_i_*) for each family or subfamily calculated for assessment of the prediction system are given by Eqs. (15)-(16).(15)(16)

Where, *N *is the total number of sequences, *obs(i) *is the number of sequences observed in class *i*, *p(i) *is the number of correctly predicted sequences of class.

**Figure 2:**
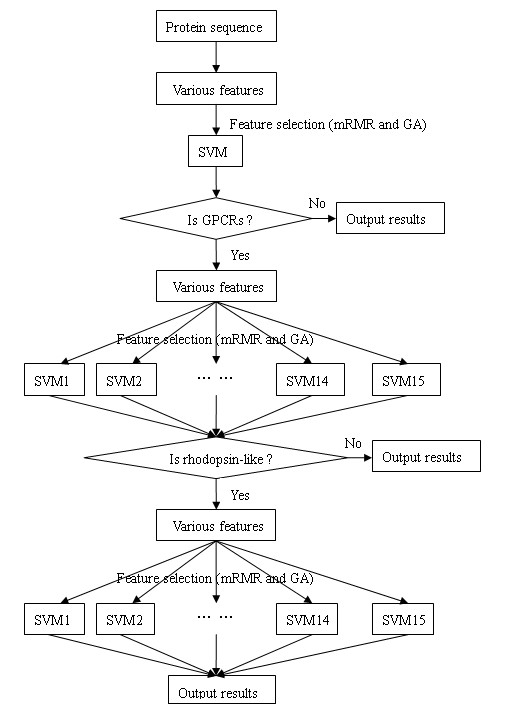
**Flowchart of the current method**.

The whole procedure for recognizing GPCRs form protein sequences and further classifying GPCRs to family and subfamily is illustrated in Figure 2, and the steps are as follows:

Step 1. Produce various feature vectors that represent a query protein sequence.

Step 2. Preselect a feature subset by running mRMR. Select an optimized feature subset from the preselect subset by GA and SVM. Predict whether the query protein belong to the GPCRs or not. If the protein is classified into non-GPCRs, stop the process and output results, otherwise, go to the next step.

Step 3. Preselect again a feature subset and further select an optimized feature subset. Predict which family the protein belongs. If the protein is divided into non-Rhodopsin like, stop the process with the output of results, otherwise, go to the next step.

Step 4. Preselect a feature subset again and select an optimized feature subset. Predict which subfamily the protein belongs to.

## Results and discussion

### Identification a GPCR from non-GPCR

**Figure 3:**
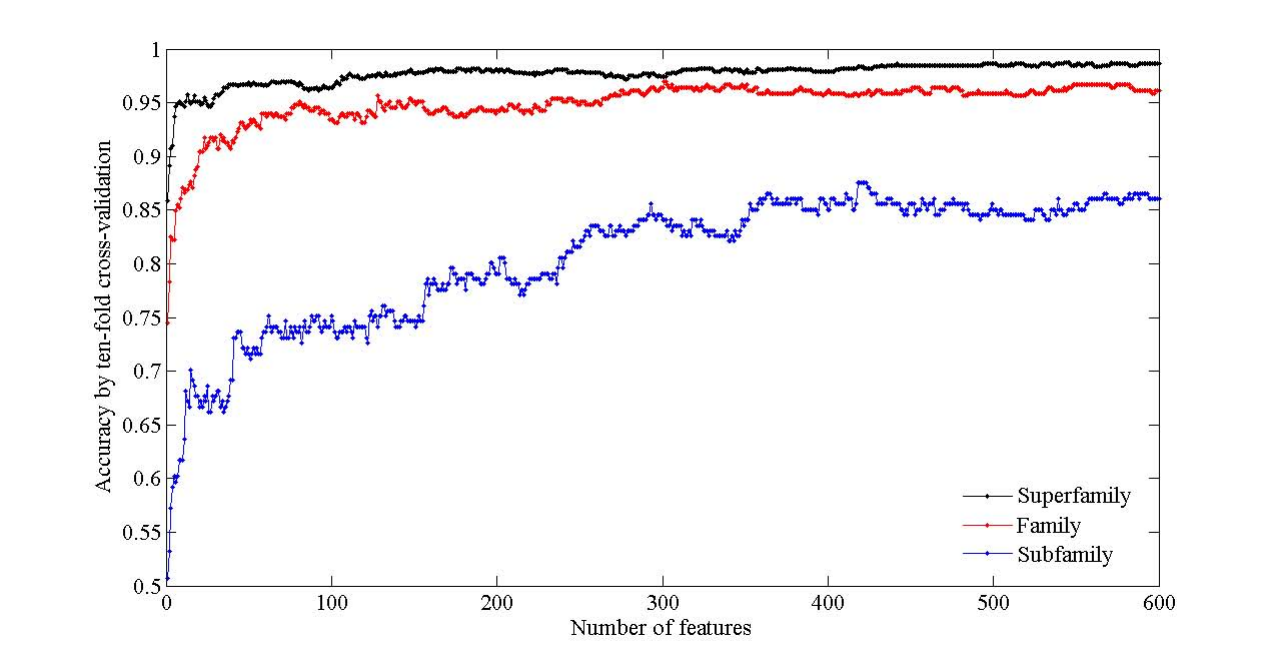
**The relationship between the accuracy and the number of features**.

At the first step of feature selection, only 600 different feature subsets are selected based on mRMR due to our limited computational power, and the feature subsets contains 1, 2, 3, ......, 598, 599 and 600 features respectively. The performance of various feature subsets for discriminating between GPCRs and other protein is investigated based on grid search for maximal 10-fold cross-validation tested accuracy with *γ *ranging among 2^-5^, 2^-4^,..., 2^15^, and *C *ranging among 2^-15^, 2^-14^,..., 2^5 ^(*γ *and *C *are needed to optimize parameters of SVM), and the results are shown in Figure 3. The accuracy for a single feature is 85.89%. And the accuracy dramatically increased when the number of features increased from 2 to 150, and achieved the highest values (98.22%) while the feature subset consists of 543 features. However, the accuracy did not change dramatically when the number of features increased to 600.

**Figure 4:**
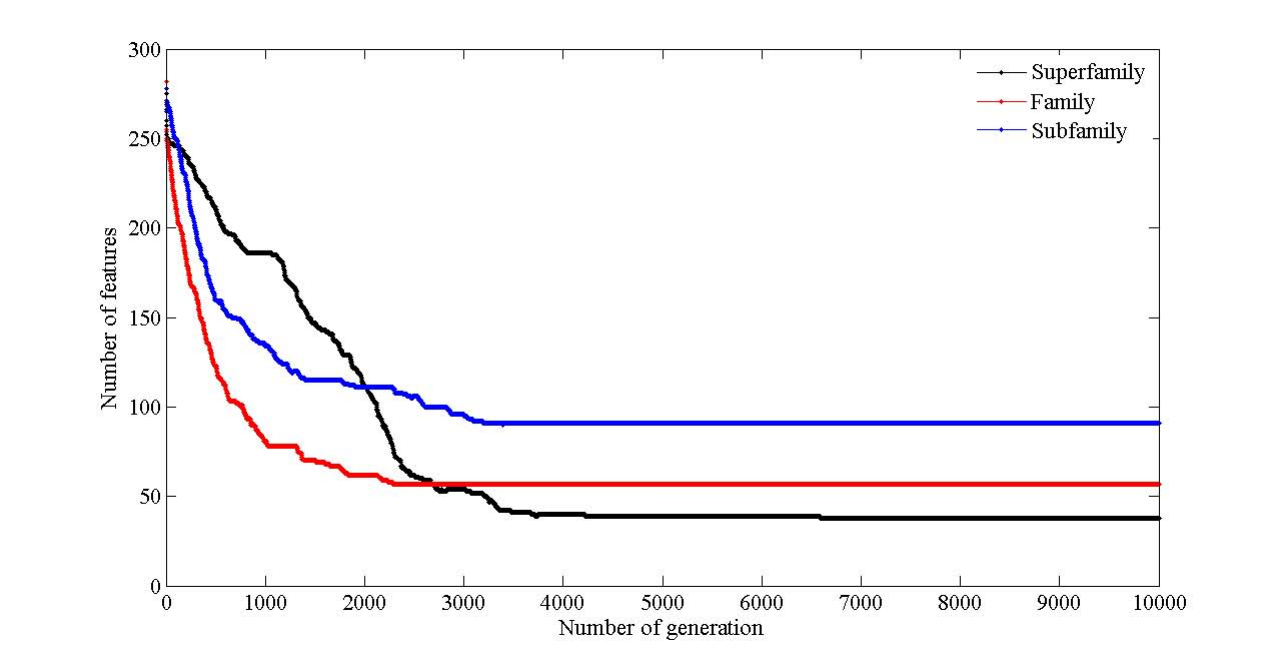
**The relationship between the number of features and the number of generations**.

**Figure 5:**
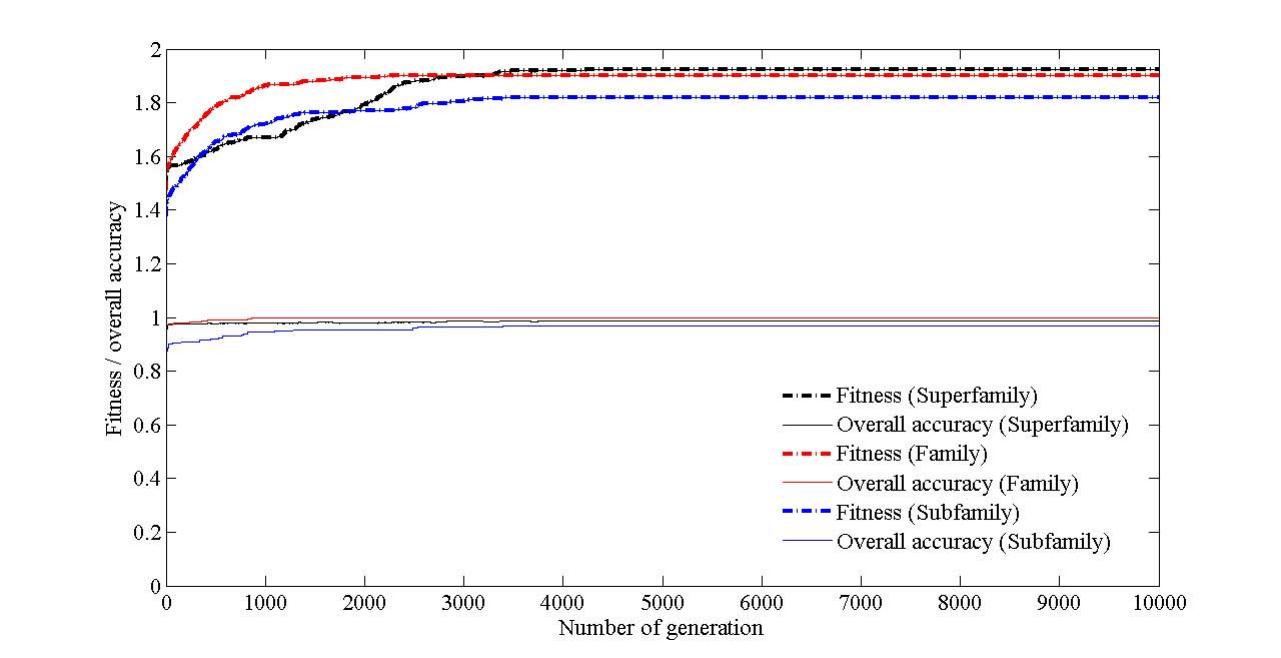
**Fitness values and overall accuracy based on the most fitted member of each generation**.

Although the highest accuracy can be obtained by using the feature subset with 543 features, many features impede the discovery of physicochemical properties that affect the prediction of GPCRs. So, we perform further GA for the preselecting feature subset that consists of 600 features. Figure 4 and Figure 5 illustrate the convergence processes for GA to select feature subset. Initially, approximate 275 features are selected by GA and a predictive accuracy about 94.93% is achieved based on 10-fold cross-validation tested. Along with the implementation of GA, the number of selected features gradually decreased while fitness is improved. Finally, the good fitness, high classification accuracy (98.77% based on 10-fold cross-validation test) and optimized feature subset (only contains 38 features) can be obtained from about 6600 generations. Consequently, the optimal classifier at superfamily level is constructed with the optimal feature subset.

**Figure 6:**
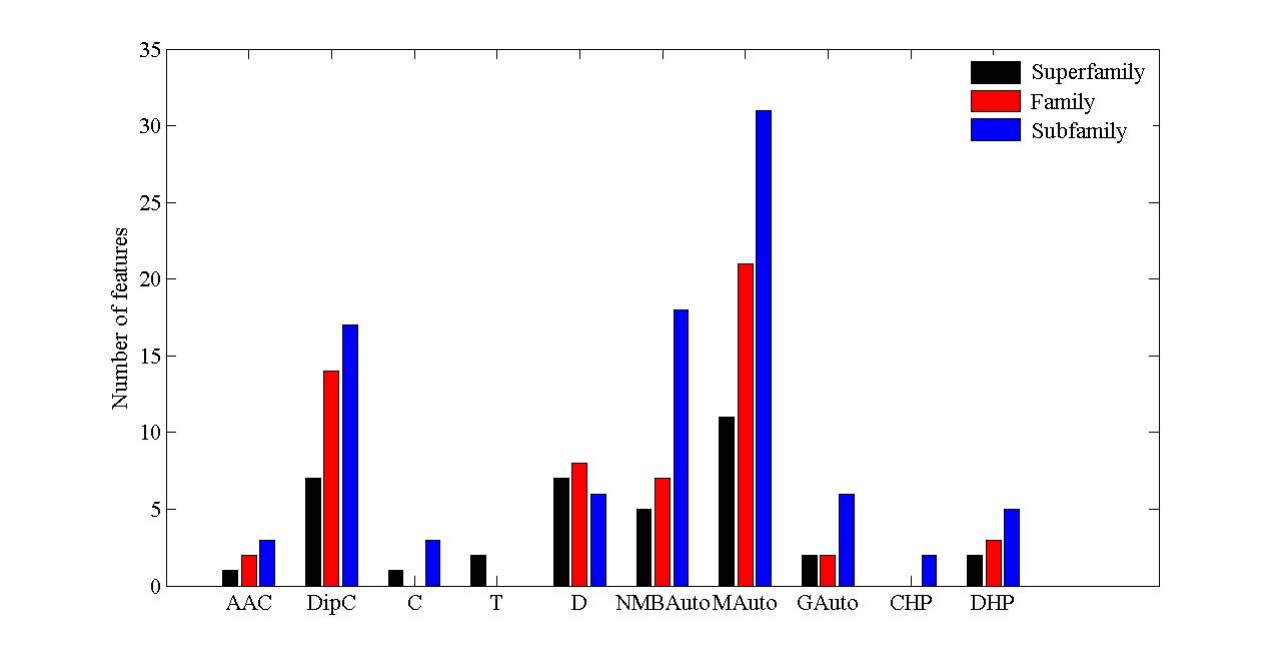
**Composition of the optimized features subset**.

The results of the optimized features subset are shown in Figure 6. The optimized features subset contains 38 features, including 1 feature of cysteine composition; 7 features of DipC based on Phe-Phe, Gly-Glu acid, His-Asp, Ile-Glu, Asn-Ala, Asn-Met and Ser-Glu; 1 feature of C based on polarity grouping; 2 features of T based on hydrophobicity and buried volume grouping; 7 features of D based on charge, hydrophobicity, Van der Waals volume, polarizability and solvent accessibility grouping; 5 features of NMBAuto based on hydrophbicity, flexibility, residue accessible surface area and relative mutability; 11 features of MAuto based on hydrophobicity, flexibility, residue volume, steric parameter and relative mutability; 2 features of GAuto based on hydrophobicity and free energy; 2 features of DHP based on pattern (*i*, *i*+*3*, *i*+*4*). The results suggest that the order of these feature groups that contributed to the discrimination GPCRs from non-GPCRs is: MAuto > Dipc and D > NMBAuto > T, GAuto and DHP > AAC and C.

### Recognition of GPCR family

Following the same steps described above, the quality of various feature subsets are investigated at family level based on grid search and 10-fold cross-validation tested. The relationship between number of feature and overall accuracy is shown in Figure 3. A significant increase in overall accuracy can be observed when the number of feature increased from 1 to 301, and the highest overall accuracy of 96.99% can be achieved.

We also further perform GA for preselecting feature subset with 600 features to acquire an optimized feature subset. The processes of optimization are displayed in Figure 4 and Figure 5. It can be observed that the number of features dramatically decreased from 250 to 57 when the number of generation increased from 1 to 2300, and the best fitness and highest overall accuracy of 99.73% can be achieved. So, the optimal classifier with 57 features is used to construct classifier at family level.

The results of the optimized feature subset are also shown in Figure 6. The optimized features subset contains 2 AAC, 14 DipC, 8 D, 7 NMBAuto, 21 MAuto, 2 GAuto and 3 DHP features. The results reveal that the order of these feature groups that contributed to the classification GPCRs into 6 families is: MAuto > DipC > D > NMBAuto > DHP > AAC and GAuto.

### Classification of GPCR subfamily

Because knowledge of GPCRs subfamilies can provide useful information to pharmaceutical companies and biologists, the identification of subfamilies is a quite meaningful topic in assigning a function to GPCRs. Therefore, we constructed a classifier at subfamily level to predict the subfamily belonging to the rhodopsin-like family. Rhodopsin-like family is considered because it covers more than 80% of sequences in the GPCRDB database [53], and the number of other family in current dataset is too few to have any statistical significance. Similarly, we also study the quality of various feature subsets from mRMR based on grid search and 10-fold cross-validation tested. The correlation between number of features and overall accuracy is also illustrated in Figure 3. Overall accuracy enhanced when the number of features increased from 1 to 300, and the highest overall accuracy of 87.56% can be obtained by using the feature subset with 418 features.

In order to get an optimized feature subset, GA is further applied to further feature selection from a preselected feature subset with 600 features. The processes of convergence are shown in Figure 4 and Figure 5. The number of features in optimized feature subset significantly decreased from 278 to 115 when the number of generation increased from 1 to 1400, and corresponding fitness value is significantly increased. Subsequently, the number of features and fitness value maintained invariable. It clearly shows a premature convergence. However, the number of features decreased from 113 to 92 when the number of generation increased from 1800 to 3100, indicating GA has ability to escape from local optima. The finally optimized feature subset with 91 features can be obtained within 3200 generations. Therefore, we developed a classifier by the features from the optimized feature subset for classifying the subfamilies of the rhodopsin-like family.

The composition of optimized feature subset is shown in Figure 6. The optimized feature subset contains 3 AAC, 17 DipC, 3 C, 6 D, 18 NMBAuto, 31 MAuto, 6 GAuto, 2 CHP and 5 DHP features. The results suggest that the order of these feature groups that contributed to the prediction subfamily belonging to the rhodopsin-like family is: MAuto > NMBAuto > DipC > D and GAuto > DHP > AAC and C > CHP.

### Comparison with GPCR-CA

**Figure 7:**
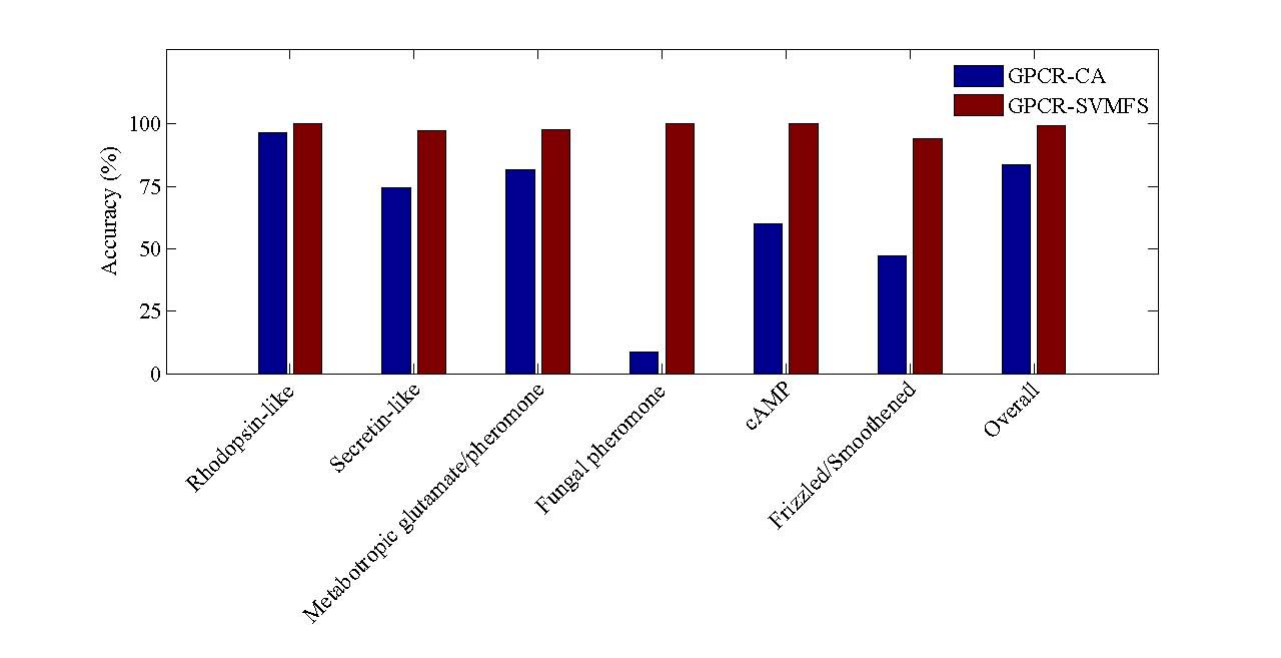
**Comparison of different method by the jackknife test at family level**.

To facilitate a comparison with GPCR-CA method developed by Xiao [10], we perform jackknife cross-validation test based on the current predictor. GPCR-CA is a two-layer classifier that is used to classify at the levels of superfamily and family, respectively, and each protein is characterized by PseAA, which is based on "cellular automation" and gray-level co-occurrence matrix factors. In the jackknife test, each protein in the dataset is in turn singled out as an independent test sample and all the rule-parameters are calculated without using this protein. The results of jackknife test obtained with proposed method in comparison with GPCR-CA are listed in Table 1 and Figure 7. The performances of the proposed predictor (GPCR-SVMFS) in predicting the subfamilies are summarized in Table 2.

**Table 1:** Comparison of different method by the jackknife at superfamily level

Method	*Acc*(%)	*Sen*(%)	*Spe*(%)	MCC
GPCR-CA [10]	91.46	92.33	90.96	N/A
GPCR-SVMFS	97.81	97.04	98.61	0.9563

**Table 2:** Success rates obtained with the GPCR-SVMFS predictor by jackknife test at subfamily level

GPCR subfamily	Number of proteins	Number of correct prediction	*Q_i_*/*Q*(%)
Amine	46	43	93.48
Peptide	72	71	98.61
Rhodopsin	17	15	88.24
Olfactory	19	19	100.0
Nucleotide	13	10	76.92
Other	34	32	94.12

Overall	201	190	94.53

It can be seen from Table 1 that the accuracy, sensitivity, specificity and *MCC *by GPCR-SVMFS are 97.81%, 97.04%, 98.61% and 0.9563, respectively, which are 4.7% to 7.6% improvement over GPCR-CA method [10]. The results indicated that the GPCR-SVMFS can identify GPCRs from non-GPCRs with high accuracy using optimized feature subset as the sequence feature.

As can be seen from Figure 7, the overall accuracy of GPCR-SVMFS is 99.18%, which is almost 15% higher than that of GPCR-CA. Furthermore, the accuracies of fungal pheromone, cAMP and frizzled/smoothened family are dramatically improved. The accuracy by GPCR-SVMFS for fungal pheromone family is 100%, approximately 93% higher than the accuracy by the GPCR-CA. The accuracies of cAMP and frizzled/smoothened are 100% and 94.12% based on GPCR-SVMFS, approximately 40% and 47% higher than the accuracy by the GPCR-CA, respectively. In additional, as for secretin and metabotropic glutamate/pheromone family, the predictive accuracies are 97.44% and 97.73% by GPCR-SVMFS, approximately 23% and 16% higher than those of GPCR-CA, indicating GPCR-SVMFS is effective and helpful for the prediction of GPCRs at family level.

As shown in Table 2, the accuracies of amine, peptide, rhodopsin, olfactory and other are 93.48%, 98.61%, 88.24% and 94.12%, respectively. Meanwhile, we also have notice that the accuracy of nucleotide is lower than that of amine, peptide, rhodopsin, olfactory, which may be caused by the less protein samples contained in nucleotide class. Although the accuracy for nucleotide is only 76.92%, the overall accuracy is 94.53% for identifying subfamiliy, indicating the current method can yield quite reliable results at subfamily level.

### Comparison with GPCRPred

Furthermore, in order to roundly evaluate our method we also performed it on another dataset used in GPCRPred [14], which is a three-layer classifier based on SVM. In the classifier, DipC is used for characterizing GPCRs at the levels of superfamily, family and subfamily. The dataset obtained from GPCRPred contains 778 GPCRs and 99 non-GPCRs. The 778 GPCRs can be divided into 5 families: 692 class A-rhodopsin and andrenergic, 56 class B-calcitonin and parathyroid hormone, 16 class C-metabotropic, 11 class D-pheromone and 3 class E-cAMP. The class A at subfamily level is composed of 14 major classes and sequences are from the work of Karchin [8].

The success rates are listed in Tables 3, 4 and 5. And the results of GPCR-SVMFS are compared with those of GPCRPred for the same dataset. From Table 3 we can see that the accuracy of GPCR-SVMFS is 0.5% higher than that of GPCRPred based on DipC at superfamily level. As can be seen from Table 4, the accuracies for class A, class B and class C are 100%, which is almost 2%, 15% and 19% higher than that of GPCRPred, respectively. Especially for the class D, the predictive accuracy is improved to 81.82% by GPCR-SVMFS, which is almost 45% higher than that of GPCRPred. As can be seen in Table 5, the accuracies of the nucleotide, viral and lysospingolipids are improved to 93.75%, 76.47%, 100.0%, about 8%, 43% and 42% higher than GPCRPred. Although the accuracy of cannabis is decreased from 100% to 90.91%, the overall accuracy is improved from 97.30% to 98.77%. All the results show that GPCR-SVMFS is superior to GPCRPred, which may be caused by the fact that optimized feature subset contains more information than single DipC, and therefore can enhance predictive performance significantly.

**Table 3:** The performance of GPCR-SVMFS and GPCRPred at superfamily level

Method	*Acc*(%)	*Sen*(%)	*Spe*(%)	MCC
GPCRPred [14]	99.50	98.60	99.80	0.9900
GPCR-SVMFS^a^	100.0	100.0	100.0	1.0000

^a ^In order to consistent with evaluation method of GPCRPred, 5-fold cross-validation is utilized.

**Table 4:** The performance of GPCR-SVMFS and GPCRPred at family level

Method	*Q_i_*/*Q *(%)					
	
	Class A	Class B	Class C	Class D	Class E	Overall
GPCRPred [14]	98.10	85.70	81.30	36.40	100.0	97.30
GPCR-SVMFS^a^	100.0	100.0	100.0	81.82	100.0	99.74

^a ^In order to consistent with evaluation method of GPCRPred, 2-fold cross-validation is utilized.

**Table 5:** The performance of GPCR-SVMFS and GPCRPred at subfamily level

Class A subfamilies	Number of proteins	*Q_i_*/*Q *(%)	
		
		**GPCRPred **[14]	GPCR-SVMFS^a^
Amine	221	99.10	100.0
Peptide	381	99.70	99.21
Hormone	25	100.0	100.0
Rhodopsin	183	98.90	99.45
Olfactory	87	100.0	100.0
Prostanoid	38	100.0	100.0
Nucleotide	48	85.40	93.75
Cannabis	11	100.0	90.91
Platelet activating factor	4	100.0	100.0
Gonadotrophin releasing hormone	10	100.0	100.0
Thyrotropin releasing hormone	7	85.70	85.71
Melatonin	13	100.0	100.0
Viral	17	33.30	76.47
Lysospingolipids	9	58.80	100.0
Overall	1054	97.30	98.77

^a ^In order to consistent with evaluation method of GPCRPred, 2-fold cross-validation is utilized.

### Predictive power of GPCR-SVMFS

In order to test the performance of GPCR-SVMFS to identify orphan GPCRs, a dataset (we called it as "deorphan") containing 274 orphan proteins are collected from the GPCRDB database (released on 2006). We further verify the 274 orphan proteins by searching accession number in the latest version of GPCRDB (released on 2009). The results indicated that 8 proteins, 19 proteins and 2 proteins belong to amine, peptide and nucleotide respectively. Finally, the dataset of 29 proteins is constructed (The dataset can be obtained from Additional file 4.

The GPCR-SVMFS is able to accurately identify 13 peptides from 19 proteins, and 2 nucleotides are completed recognized. However, none of the 8 amines is correctly identified. So, overall success rate is 19/29 = 51.72%. The result is higher than that of completely randomized prediction, because the rate of correct identification by randomly assignment is 1/6 = 16.67% if the protein samples are completely randomly distributed among the 6 possible subfamilies (i.e. amine, peptide, rhodopsin, olfactory, nucleotide and other). The results imply that GPCR-SVMFS is indeed powerful to identify orphan GPCRs.

In addition, the prediction power of GPCR-SVMFS is also evaluated at family level and subfamily level by using 8 independent dataset, which are collected based on the GPCRDB (released on 2009). Three of the 8 dataset at family level are rhodopsin-like, metabotropic and secretin-like, which contains 20290, 1194 and 1484 proteins, respectively. Other 5 dataset at subfamily level are amine, peptide, rhodopsin, olfactory and nucleotide. The 5 dataset is composed of 1840, 4169, 1376, 9977 and 576 proteins, respectively (8 dataset are given in Additional file 5, 6, 7, 8, 9, 10, 11, 12).

The results at family level are shown in Table 6. The proposed method achieves accuracy of 96.16% for rhodopsin-like, 85.76% for metabotropic and 68.53% for secretin-like, and an overall accuracy of 93.81% can also be obtained. The results indicate that the performance of GPCR-SVMFS is good enough at family level.

**Table 6:** The prediction power of GPCR-SVMFS to independent dataset at family level

GPCR family	Number of proteins	Number of correct prediction	*Q_i_*/*Q*(%)
Rhodopsin-like	20290	19510	96.16
Metabotropic	1194	1024	85.76
Secretin-like	1484	1017	68.53
Overall	22972	21551	93.81

The results for 5 subfamilies are listed in Table 7. The prediction accuracies for the rhodopsin, amine and peptide reach 87.79%, 80.22% and 74.12%, respectively. For the largest subfamily (olfactory) that contains 9977 proteins, the accuracy achieves the highest values of 90.96%. Although the accuracy for nucleotide is only 54.69%, the overall prediction accuracy achieves 84.54% for classifying subfamily, indicating the GPCR-SVMFS method can yield good results at subfamily level.

**Table 7:** The prediction power of GPCR-SVMFS to independent dataset at subfamily level

GPCR subfamily	Number of proteins	Number of correct prediction	*Q_i_*/*Q*(%)
Amine	1840	1476	80.22
Peptide	4169	3090	74.12
Rhodopsin	1376	1208	87.79
Olfactory	9977	9075	90.96
Nucleotide	576	315	54.69
Overall	17938	15164	84.54

## Conclusion

With the rapid increment of protein sequence data, it is indispensable to develop an automated and reliable method for classification of GPCRs. In this paper, a three-layer classifier is proposed for GPCRs by coupling SVM with feature selection method. Compared with existing methods, the proposed method provides better predictive performance, and high accuracies for superfamily, family and subfamily of GPCRs in jackknife cross-validation test, indicating the investigation of optimized features subset are quite promising, and might also hold a potential as a useful technique for the prediction of other attributes of protein.

## Authors' contributions

ZCL conceived the idea and developed the programs, carries out the analyses and drafted the manuscript. XZ contributed to the ideas on overall design, implementation. ZD carried out data acquisition, guided the implementation of the work. XYZ supervised the design of the system, and advised on the manuscript preparation. All authors read and approved the final manuscript.

## Supplementary Material

Additional file 1 **Eight amino acid properties extracted from AAIndex database are selected to compute autocorrelation features**. Click here for file

Additional file 2 **The values of seven properties are obtained from AAIndex database, and the definitions of amino acids attributed to each group are shown in the table**. Click here for file

Additional file 3 **More explain and an example to compute probability**. Click here for file

Additional file 4 **Deorphan dataset**. The file contains orphan protein's Swiss-Prot accession number and its corresponding sequence. Click here for file

Additional file 5 **Rhodopsin-like dataset**. The file contains rhodopsin-like protein's Swiss-Prot accession number and its corresponding sequence. Click here for file

Additional file 6 **Metabotropic dataset**. The file contains metabotropic protein's Swiss-Prot accession number and its corresponding sequence. Click here for file

Additional file 7 **Secretion-like dataset**. The file contains secretion-like protein's Swiss-Prot accession number and its corresponding sequence. Click here for file

Additional file 8 **Amine dataset**. The file contains amine protein's Swiss-Prot accession number and its corresponding sequence. Click here for file

Additional file 9 **Peptide dataset**. The file contains peptide protein's Swiss-Prot accession number and its corresponding sequence. Click here for file

Additional file 10 **Rhodopsin dataset**. The file contains rhodopsin protein's Swiss-Prot accession number and its corresponding sequence. Click here for file

Additional file 11 **Olfactory dataset**. The file contains olfactory protein's Swiss-Prot accession number and its corresponding sequence. Click here for file

Additional file 12 **Nucleotide dataset**. The file contains nucleotide protein's Swiss-Prot accession number and its corresponding sequence. Click here for file
