# Supplementary material for: Classification of G-protein coupled receptors based on support vector machine with maximum relevance minimum redundancy and genetic algorithm
Source: BMC Bioinformatics. 2010 Jun 16;11:325. doi: 10.1186/1471-2105-11-325 (PMC2905366; doi:10.1186/1471-2105-11-325)
Supplement: Additional file 1 — Eight amino acid properties extracted from AAIndex database are selected to compute autocorrelation features. [file 1471-2105-11-325-S1.doc]

Table 1. Eight physicochemical properties used in autocorrelation features

| Properties description | Entry | Reference |
| --- | --- | --- |
| Hydrophobicity | [CIDH920105](http://www.genome.ad.jp/dbget-bin/www_bget?aaindex+CIDH920105) | Cid et al., 1992 |
| Flexibility | BHAR880101 | Bhaskaran-Ponnuswamy, 1988 |
| Polarizability | CHAM820101 | Charton-Charton, 1982 |
| Free energy | CHAM820102 | Charton-Charton, 1982 |
| Residue accessible surface area | CHOC760101 | Chothia, 1976 |
| Residue volume | [BIGC670101](http://www.genome.ad.jp/dbget-bin/www_bget?aaindex+BIGC670101) | Bigelow, 1967 |
| Steric parameter | CHAM810101 | Charton, 1981 |
| Relative mutability | DAYM780201 | Dayhoff et al., 1978 |
