# Supplementary material for: Classification of G-protein coupled receptors based on support vector machine with maximum relevance minimum redundancy and genetic algorithm
Source: BMC Bioinformatics. 2010 Jun 16;11:325. doi: 10.1186/1471-2105-11-325 (PMC2905366; doi:10.1186/1471-2105-11-325)
Supplement: Additional file 2 — The values of seven properties are obtained from AAIndex database, and the definitions of amino acids attributed to each group are shown in the table. [file 1471-2105-11-325-S2.doc]

Table 2. Attributes and division of the amino acids

| Properties description | Entry | Divisions | | |
| --- | --- | --- | --- | --- |
| Charge | KLEP840101 | Positive | Neutral | Negative |
|  |  | K R | A N C Q G H I L M F P S T W Y V | D E |
| Hydrophobicity | JURD980101 | Polar | Neutral | Hydrophobicity |
|  |  | R N D Q E K | G A S T P H Y | C L V I M F W |
| Van der Waals volume | FAUJ880103 | range 0-2.78 | range 2.95-4 | range 4.03-8.08 |
|  |  | G A S T P D C | N V E Q I L | R H K M F W Y |
| Polarity | GRAR740102 | range 4.9-6.2 | range 8.0-9.2 | range 10.4-13.0 |
|  |  | L I F W C M V Y | P A T G S | H Q R K N E D |
| Polarizability | CHAM820101 | range 0-0.108 | range 0.128-0.186 | range 0.219-0.409 |
|  |  | G A S D T | C P N V E Q I L | K M H F R Y W |
| Buried volumes | HARY940101 | range 63.8-120 | range 120-190 | range 190-231 |
|  |  | A D C G S T | N H I L K M P V Q E | R F W Y |
| Solvent Accessibility | FAUJ880109 | Buried | Exposed | Intermediate |
|  |  | A L F C G I V W | P K Q E N D | M P S T H Y |
