# Supplementary material for: Classification of G-protein coupled receptors based on support vector machine with maximum relevance minimum redundancy and genetic algorithm
Source: BMC Bioinformatics. 2010 Jun 16;11:325. doi: 10.1186/1471-2105-11-325 (PMC2905366; doi:10.1186/1471-2105-11-325)
Supplement: Additional file 3 — More explain and an example to compute probability. [file 1471-2105-11-325-S3.doc]

For example, we want to calculate the mutual information between feature X=[1 2 1 2 1] and classification variable L=[2 1 2 1 1].

The joints of X and L are: 1—2, 2—1, 1—2, 2—1, 1—1.

Through tallying the samples of categorical variables in the data, we can obtain:

p(X_1_,L_1_)=1/5=0.2 (The joints of 1—1 happens only 1 time in the 5 joints of 1—2, 2—1, 1—2, 2—1, 1—1.)

p(X_1_,L_2_)=2/5=0.4 (The joints of 1—2 happens only 2 times in the 5 joints of 1—2, 2—1, 1—2, 2—1, 1—1.)

p(X_2_,L_1_)=2/5=0.4 (The joints of 2—1 happens only 2 times in the 5 joints of 1—2, 2—1, 1—2, 2—1, 1—1.)

p(X_2_,L_2_)=0/5=0 (The joints of 2—2 happens no time in the 5 joints of 1—2, 2—1, 1—2, 2—1, 1—1.)

p(X_1_)=3/5=0.6

p(X_2_)=2/5=0.4

p(L_1_)=3/5=0.6

p(L_2_)=2/5=0.4

Where, X_1_ = 1, X_2_=2, L_1_=1, L_2_=2.

p(X_1_) is frequency of feature values 1 in vector X.

p(X_2_) is frequency of feature values 2 in vector X.

p(L_1_) is frequency of classification variable 1 in vector L.

p(L_2_) is frequency of classification variable 2 in vector L.

p(X_1_,L_1_) is joint frequency of feature values 1 and classification variable 1.

p(X_1_,L_2_) is joint frequency of feature values 1 and classification variable 2.

p(X_2_,L_1_) is joint frequency of feature values 2 and classification variable 1.

p(X_2_,L_2_) is joint frequency of feature values 2 and classification variable 2.

So,

=p(X_1_,L_1_)*log_2_(p(X_1_,L_1_)/ (p(X_1_)* p(L_1_)))+

p(X_1_,L_2_)*log_2_(p(X_1_,L_2_)/ (p(X_1_)* p(L_2_)))+

p(X_2_,L_1_)*log_2_(p(X_2_,L_1_)/ (p(X_2_)* p(L_1_)))

=0.2*log_2_(0.2/(0.6*0.6))+

0.4*log_2_(0.4/(0.6*0.4))+

0.4*log_2_(0.4/(0.4*0.6))

= 0.42
